# A “Cultural” Renaissance: Genomics Breathes New Life into an Old Craft

**DOI:** 10.1128/mSystems.00092-19

**Published:** 2019-05-07

**Authors:** Paul Carini

**Affiliations:** a Department of Soil, Water and Environmental Science, University of Arizona, Tucson, Arizona, USA

**Keywords:** cultivation, functional genomics, microbial metabolism, microbial physiology, uncultured microbes

## Abstract

Sometimes, to move ahead, you must take a look at where you have been. Culturing microbes is a foundational underpinning of microbiology. Before genome sequencing, researchers spent countless hours tediously deducing the nutritional requirements of bacterial isolates and tinkering with medium formulations to entice new microbes into culture. This art of cultivation took a back seat to the powerful molecular tools of the last 25 years, and as a result, many researchers have forgotten the utility of having a culture in hand. This perception is changing, as there is clearly a renewed interest in isolating microbes from various environments. Here, I suggest three focus areas to ensure continued growth and success of this “cultural” renaissance, including (i) setting clear cultivation goals, (ii) funding exploratory cultivation, and (iii) culturing and studying unusual organisms. “Unculturable” is a frame of mind, not a state of microbiology; it is time to dust off the bottle of yeast extract.

## Abstract (precis)

Sometimes, to move ahead, you must take a look at where you have been. Culturing microbes is a foundational underpinning of microbiology.

## PERSPECTIVE

For over a century, our understanding of microbial biology has been predicated on the ability of scientists to cultivate and study organisms under controlled laboratory conditions. Indeed, until the 1980s, our knowledge of microbial diversity was constrained to those microbes that grew in the laboratory or appeared under the microscope. Yet, it was also understood that the majority of microbes were recalcitrant to growth in the laboratory. The incongruence between enumeration by direct microscopic counting and culturable counting was eloquently presented by Staley and Konopka as “the great plate count anomaly” (1) and is oft attributed to the widespread notion that more than 99% of bacteria are unculturable. This anomaly was confirmed when Pace and colleagues started investigating natural environments with molecular tools targeting rRNA genes, lifting the veil masking vast uncultured microbially diverse populations for the first time (2). Perhaps inadvertently, the coincident timing of the great plate count anomaly and Pace et al.’s early rRNA-based approaches became the impetus for a major shift in the ways that microbial ecologists explored natural microbial assemblages; a whole generation of researchers traded piles of agar plates and growth curves for DNA sequencers, computer servers, and metabolic reconstructions from genomic data. Today, DNA sequencing is perceived (or at least touted) as the way to circumvent the problem of microbial unculturability.

## A “CULTURAL” RENAISSANCE

**Sooner or later, everything old is new again.**

—Stephen King (29)

After a decade of refining the “big-data” side of microbiology, we are in the midst of a cultural renaissance in which the importance of cultivation-focused efforts has been rediscovered, with exciting results and implications. Several groups have demonstrated that a high percentage of host-associated microbiome members can be cultivated (3–7) and that such culture collections are indispensable for deducing important aspects of microbiome function (8–10). The renewed motivation to culture cells from nonhost environments, such as soil (11), aquatic environments (12), and the deep biosphere (13), is inspired, in part, by these successes and the enormous amounts of molecular data that have illuminated seemingly limitless microbially diverse populations that we know very little about (14). We can now dissect life’s blueprints for these uncultivated lineages and use this information to facilitate cultivation, insight not afforded to previous generations of researchers.

Translating genomic information into an understanding of organismal physiology is anything but straightforward, and culturing environmental microbes is very time-intensive. This is highlighted by the story of the abundant marine heterotroph *Pelagibacter* (SAR11). *Pelagibacter* was discovered with molecular methods in 1990 (15) and cultured on natural seawater medium 12 years later (16). Surprisingly, it was not immediately obvious from *Pelagibacter* genome sequences (17) what nutrients were required for growth. In fact, with genomes in hand, it took an additional 8 years plus several postdocs and graduate students to translate that information into a defined medium (reference 18 and references therein). Subsequent laboratory studies have elucidated the unusual biology that contributes to *Pelagibacter*’s unparalleled success in the sea and its key role in Earth’s biogeochemistry (reviewed in reference 19). The unique characteristics of *Pelagibacter* cells were discovered by careful testing in the laboratory after scrutinizing of genomes, transcriptomes, and proteomes, a back-and-forth process that requires a culture, a genome, and patience.

*Pelagibacter*’s narrative highlights an important lesson. By shying away from the challenge of studying microbial cultures, we effectively turn our backs on an entire world of emergent properties that govern microbial activity and ecosystem function, properties that are not always predictable *a priori* from sequence information. To analogize, one cannot understand the experience of driving a Ferrari from the list of its components; a parts list does not convey the handling, the sound, or the driver’s connection with the machine. Because of the investment in sequencing technology, our genetic inventories are more extensive than ever, yet the cultivation of novel microbes remains a complex task, much like assembling a 3-dimensional puzzle. Hypotheses generated from genome-based metabolic reconstructions from single cells or metagenomes provide a crucial dimension to guide the assembly of the puzzle, but genomes are a parts list; we seek an understanding of the emergent principles of the cells themselves and the communities that they constitute.

While this cultural renaissance is indicative of a greater awareness for the need to study cell cultures to comprehensively understand Earth’s microbiome, it is not always clear how to leverage and integrate molecular data to do that. Below are three steps that will lay a foundation for the future of integrated Earth microbiome research with an emphasis on elucidating the uncultured microbially diverse populations that we understand the least.

## DECIDE WHAT TO CULTURE

Let us be clear; from a sheer numbers perspective, it is impossible to culture every microbe on Earth no matter what the environment. Because of this, we should set cultivation goals that are specific, achievable, and relevant. I envision explicit most-wanted lists that use molecular data to inform exactly which taxa we should target for cultivation and why they are important. Further, this molecular data can be used to identify potential cultivation strategies through metabolic reconstruction. The rationales for cultivation of a particular taxa will vary across research groups but may include (i) an organism’s high relative abundance, (ii) its key role in biogeochemistry or bioremediation, (iii) its potential to produce natural products, and (iv) its substantial divergence from cultured taxa. Table 1 contains a short list of potential most-wanted microbes from various ecosystems that would be valuable to obtain in culture, based on an informal survey of colleagues and my own interests.

**TABLE 1:** Examples of microbes that are most wanted in culture

Candidate organism or group	Environment(s)	Phylum	Reason for cultivation
SAR202	Marine	*Chloroflexi*	Abundant in mesopelagic waters
SAR86	Marine	*Gammaproteobacteria*	Abundant in surface waters
SAR324	Marine	*Deltaproteobacteria*	Ubiquitous in the dark ocean
“*Candidatus* Actinomarinidae”	Marine	*Actinobacteria* (OM1)	Streamlined genome and key role in biogeochemistry
“*Candidatus* Thalassoarchaea”	Marine	*Euryarchaeota* (Marine Group II)	Abundant and key role in biogeochemistry
“*Candidatus* Marinimicrobia”	Marine	Candidate phylum marine group A	Abundant and key role in biogeochemistry
Water column B *Thaumarchaeota*	Marine	*Thaumarchaeota*	Key role in biogeochemistry
Marine group III *Euryarchaeota*	Marine	*Euryarchaeota*	Deep mesopelagic and bathypelagic communities
“*Candidatus* Udaeobacer copiosus”	Soil	*Verrucomicrobia*	Numerically dominant in some soils
*Acidobacteriia* spp.	Soil	*Acidobacteria*	Potential secondary metabolite producer
*Blastocatellia* spp.	Soil	*Acidobacteria*	Potential secondary metabolite producer
*Holophagales* spp.	Soil	*Acidobacteria*	Potential secondary metabolite producer
“*Candidatus* Rokubacteria”	Soil	Candidate phylum Rokubacteria (SPAM)	Novel phylum
“*Candidatus* Dormibacter”	Soil	Candidate phylum AD3	Novel phylum
“*Candidatus* Eremiobacteraeota”	Soil	Candidate phylum WPS-2	Novel phylum
Anaerobic methanotroph (ANME) clades 1, 2, and 3	Sediment	*Euryarchaeota*	Key role in biogeochemistry
Bathyarchaeota	Sediment	*Crenarchaeota*	Key role in biogeochemistry
“*Candidatus* Atribacteria”	Sediment	Candidate phylum Atribacteria (OP9/JS1)	Key role in biogeochemistry
Assorted *Chloroflexi*	Assorted	*Chloroflexi*	Contaminant remediation and key roles in biogeochemistry
“*Candidatus* Asgardarchaeota”	Assorted	Candidate superphylum Asgardarchaeota	Novel phylum key to eukaryote origins
Any representative of the Candidate Phylum Radiation	Assorted	Assorted	Divergent from all cultured bacteria

## INVEST IN RISKY CULTURE-BASED WORK

A popular perception is that exploratory culture-based research is unusually risky and unlikely to result in new isolates of interest. First, this is false (4, 11, 12, 20, 21). Second, all science is risky, and much is unsuccessful; how much do we want to learn? While it is tempting to point the finger at funding agencies for not supporting exploratory cultivation work, it is our colleagues, the reviewers and panelists, that marginalize these proposals. Funding agencies could shift this discourse by soliciting proposals that explicitly aim to (i) coordinate exploratory cultivation experiments with creative genomics or metabolomics approaches, (ii) develop new or higher-throughput cultivation strategies, and (iii) support long-term projects that investigate the biology of slow-growing or noncanonical model organisms that do not conform to the scale of traditional funding cycles. As J. Cameron Thrash explores in a companion Perspective in this issue, we need to constrain the costs of investing in cultivation-based work. If microbes hold the answers to many solvable problems, at some point we will need to invest in culturing them. Long-term, I envision well-funded high-throughput cultivation core labs that culture bacteria from user samples. Not only would these centers preserve living biodiversity that will almost certainly have utility in a changing world, but the cultivation of enigmatic taxa might be a strict numbers game: the more experiments that are conducted, the more likely they will capture something novel.

## BUILD KNOWLEDGE BRIDGES WITH CULTURES

The ability to sequence DNA is no longer a limiting factor in understanding microbial communities; the bottleneck lies with translating these sequence data into a functional context. The root of this limitation is that most genes have poor or no functional annotation, and our interpretation of the remainder is biased by the physiology of a few model organisms, the so-called streetlight effect (22). Isolating and studying organisms with unusual biology can bridge this knowledge gap. For example, focused functional genomics approaches uncovered widespread lipid remodeling in marine heterotrophs and related this process to poorly annotated genes (23–25). Likewise, the genome-facilitated discovery of complete ammonia oxidation to nitrate by *Nitrospira* cultures fundamentally changed our understanding of the nitrogen cycle (26). Moreover, high-throughput functional genomics approaches, such as transposon insertion sequencing (TnSeq), have proven to be scalable and powerful for identifying the fitness landscape of poorly annotated genes (27). While these approaches certainly require more effort than sequencing alone, the information that they provide in many cases unambiguously links genotype to phenotype.

## OLIGOTROPHY AS AN EMERGENT PRINCIPAL

The challenge of translating genomic data into microbial cultures and linking genes with function is a driving force for our research group. However, an important dimension to the problem of isolating uncultured microbes may be an emergent principle not easily deduced from genomes: oligotrophy. Oligotrophy describes the paradoxical and enigmatic phenomenon of microbial cells growing optimally when nutrient availability is low. New evidence suggests that the numerically abundant microbes in nonhost systems are oligotrophs (28). Yet, we have a massively incomplete understanding of the physiological basis for oligotrophy and how oligotrophs contribute to microbial community stability and ecosystem function.

We have built our lab around the idea that many uncultured soil microbial lineages are oligotrophs. In general, oligotrophs are challenging to culture because they are small, slow-growing cells and do not attain high yields on low nutrient media, rendering common methods of quantifying microbial growth ineffective. To circumvent this, we invested in equipment to separate cells from environmental matrices and count oligotrophic cultures with high throughput and sensitivity. Our initial cultivation results are encouraging; across several experiments, we have ∼3,000 cultures, some of which are representatives of uncultivated lineages of important soil bacteria. In these experiments, the concentration of a defined set of nutrients significantly influenced what grew, and many strains isolated on low-nutrient medium appear to be obligate oligotrophs that are inhibited by modest concentration increases of the same nutrients. Our long-term goal is to increase the efficiency and reduce the person-hour cost of cultivation by automating certain aspects of the cultivation process so that we can spend more time investigating interesting isolates in-depth.

My hopes are not only that the cultural renaissance is perceived as a reference to a renewed interest in culturing cells but also that it invokes a cultural shift in the way microbiologists perceive cells and their role in modern microbiology. Cells are not simply “bags of biochemistry” but living entities that are seamlessly integrated into the physical, ecological, and evolutionary landscape of Earth. Because of this, the methods by which we choose to study them must be similarly integrated.
